# Transcranial Magnetic Stimulation in the Differential Diagnosis of Unilateral Peripheral Facial Nerve Palsy

**DOI:** 10.3390/brainsci13040624

**Published:** 2023-04-06

**Authors:** Vera E. A. Kleinveld, Sarah Platzgummer, Julia Wanschitz, Corinne G. C. Horlings, Wolfgang N. Löscher

**Affiliations:** Department of Neurology, Medical University Innsbruck, 6020 Innsbruck, Austria; sarah.platzgummer@student.i-med.ac.at (S.P.); julia.wanschitz@i-med.ac.at (J.W.); corinne.horlings@tirol-kliniken.at (C.G.C.H.); wolfgang.loescher@i-med.ac.at (W.N.L.)

**Keywords:** facial nerve, facial palsy, Bell’s palsy, transcranial magnetic stimulation, nerve conduction study

## Abstract

(1) Background: This study aims to assess the diagnostic accuracy of parameters based on a combination of transcranial magnetic stimulation (TMS) and electrical stimulation (ES) in the differentiation between idiopathic and secondary facial palsy in a large cohort of patients. (2) Methods: Patients with unilateral facial palsy ≤7 days after symptom onset were included. Compound muscle action potential (CMAP) amplitudes were measured after stimulation of both facial nerves at (A) the internal acoustic meatus using TMS, CMAP-TMS, and (B) at the stylomastoid foramen using electrical stimulation, CMAP-ES. To express the degree of nerve dysfunction in the facial canal specifically, the amplitude reduction of the CMAP-TMS in relation to CMAP-ES was calculated and expressed as a percentage (amplitude reduction over the facial canal, ARFC). Receiver Operating Characteristic (ROC) curves were constructed to assess the diagnostic accuracy of ARFC as a marker to discriminate between patients with idiopathic and secondary facial palsy. (3) Results: Data from 498 patient records were analyzed. Idiopathic facial palsy was diagnosed in 424 patients, and secondary facial palsy in 74 patients. The area under the ROC curve for ARFC was 0.398. (4) Conclusions: The overall diagnostic accuracy of this method to differentiate secondary from idiopathic facial palsy is low.

## 1. Introduction

With an incidence of 7–40 per 100,000, peripheral facial palsy is the most common cranial mononeuropathy. Common etiologies for secondary facial palsy include infections (e.g., neuroborreliosis or zoster oticus), trauma, neoplastic processes, diabetes mellitus and autoimmune disorders, and contribute to 25–40% of all peripheral facial palsies [1]. The pathophysiology of secondary facial palsy is dependent on the cause: inflammation causing ischemic damage and structural abnormality or trauma disrupting the relationship between the facial nerve and its bony canal have been proposed [2]. These changes ultimately lead to axonal damage with Wallerian degeneration. When no secondary cause can be identified, peripheral facial palsy is diagnosed as idiopathic [3], which is assumed to be a demyelinating lesion [4]. As treatments differ considerably, a fast, reliable and easily accessible method to differentiate idiopathic from secondary facial palsy is needed.

Current clinical practice depends on local standard operating procedures, including neuroimaging, cerebrospinal fluid (CSF) analysis, neurophysiological testing and/or clinical follow-up. CSF analysis, which is normal in idiopathic facial palsy, is used to rule out infectious etiologies [5,6]. When a non-infectious etiology is suspected, magnetic resonance imaging (MRI) of the cerebrum can be used to detect structural pathologies, while computed tomography (CT) of the anterior cranial fossa is used when osseous lesions are suspected [7]. However, routine diagnostic imaging use is not considered contributive in suspected idiopathic facial palsy [4].

Neurophysiological examinations to differentiate idiopathic from secondary facial palsy consist of a combination of transcranial magnetic stimulation (TMS) and electrical stimulation (ES) and recording of the compound muscle action potential (CMAP) amplitude of a facial muscle. This is based on the assumption that idiopathic facial nerve palsy results from nerve compression and edema within the facial canal [4]. As TMS stimulates the nerve proximal of the facial canal while ES stimulates the nerve distal of the facial canal, an amplitude reduction of the CMAP after magnetic stimulation (CMAP-TMS) in relation to the CMAP amplitude after electric stimulation (CMAP-ES) allows for the identification of conduction disorders in the intracanalicular part of the facial nerve. This is believed to be indicative of idiopathic facial nerve palsy. However, previous studies have yielded conflicting results [8,9,10,11,12]. In addition, the motor response might be related to the clinical severity of the palsy [13,14].

Based on the largest cohort of patients with new-onset unilateral facial palsy to date, this study aims to clarify whether the combination of ES and TMS can reliably differentiate idiopathic from secondary facial palsy.

## 2. Materials and Methods

### 2.1. Patient Inclusion and Follow-Up

This study was approved by the Medical Ethics Committee of the Medical University Innsbruck (K Nr: 1322/2021).

This retrospective study included only patients with a new-onset unilateral peripheral facial palsy (ICD 10: G51) who presented to the Department of Neurology, Medical University Innsbruck (Austria). Clinical and electrophysiological data were collected. Only patients who underwent full electrophysiological examination within 7 days after symptom onset and completed the one-week follow-up were included. We excluded patients with absolute contra-indications for ES or TMS of the facial nerve (history of seizures and presence of intracranial hardware) and patients with sparing of the upper face musculature, suggestive of central facial palsy due to a corticobulbar etiology. Patients were recruited from 1 January 2005 to 30 June 2021.

Facial nerve palsies were either classified as idiopathic or secondary. The classification was based on clinical work-up and a follow-up visit one week after presentation, performed by neurologists. Routine blood tests were part of the diagnostic work-up. Based on clinical and laboratory findings, patients with suspected secondary etiologies underwent CSF analysis, MRI-cerebrum or CT-cerebrum. 

When the initial work-up did not show any abnormalities or symptoms progression or improvement at follow-up, the palsy was classified as idiopathic.

The severity of the palsy was graded on a six-point scale according to the House and Brackmann (HB) classification system [15].

### 2.2. Electrophysiological Examinations

Electrophysiological examinations were performed within 7 days after symptom onset. The responses after percutaneous ES and TMS were measured as the negative peak of the CMAP amplitude. CMAP was recorded by an electrode (Genuine Grass^®^ Gold Cup electrode, 10 mm diameter) over the musculus nasalis, as the CMAP of this muscle shows a clearly defined onset [12]. The recording electrode was placed on the ipsilateral side of the nose, while the indifferent electrode was placed on the bridge of the nose.

For ES, a Nicolet Viking™ EDX electrical stimulator was used to excite the post-canalicular segment of the facial nerve (0.2 ms stimulus duration) at the site of the stylomastoid foramen. The first stimulation was performed with approximately 10 mA and was increased to supramaximal stimulation. To excite the facial nerve at the facial canal entry zone, a MagStim™ 200 magnetic stimulator was used for single pulse stimulation with 82 μs pulse-width. The diameter of the stimulation coil was 90 mm, and it was positioned parieto-occipitally behind the ear, positioned with an anti-clockwise current orientation on the right side and a clockwise current orientation on the left. The first stimulation was performed with an output of approximately 30% of the maximum stimulator output percentage and was increased until the CMAP amplitude did not increase at two subsequent stimulations. For TMS and ES, two recordings at maximum output were superimposed to establish reproducibility. All recordings were performed on both sides. The clinically healthy side was examined first.

### 2.3. Data analysis and Statistics

Shapiro–Wilk tests were performed to test the normality of the data. CMAP-TMS and CMAP-ES were used to calculate the amplitude reduction over the facial canal (ARFC). ARFC was calculated as 100—(CMAP-TMS/CMAP-ES * 100%).

Delta amplitude reduction (ΔARFC) was calculated as the absolute difference in percentage of the ARFC on the healthy and affected side. To assess the distal function of the nerve, the CMAP-ES on the affected side was expressed as percentage of that of the healthy side. In case any of the CMAP amplitudes was 0, an amplitude of 0.01 was registered to be able to perform calculations involving proportions.

Receiver operating characteristics (ROC) curves were generated, and areas under the curves (AUCs) were calculated for ARFC and ΔARFC. Measures of facial nerve function were correlated with disease duration and severity.

We used Mann-Whitney U tests to compare outcomes between two independent groups, Fisher’s exact test to compare frequencies between groups and Spearman’s rank test for non-parametric correlation analysis. *p* < 0.05 was set as statistically significant. Results are presented as mean and standard deviation if not stated otherwise. All statistical analyses were performed using IBM^®^ SPSS (version 28.0.1.0 (142)). 

## 3. Results

### 3.1. Demographics and Clinical Characteristics

The final analysis included 498 patients with new-onset unilateral facial palsy. Of those, 74 patients were diagnosed with secondary facial palsy. Non-infectious etiologies included diabetes (*n* = 44), Guillain-Barré syndrome (*n* = 2), Melkerson-Rosenthal syndrome (*n* = 1), polyradiculitis cranialis (*n* = 5), or tumors compressing the facial nerve (*n* = 3). Infectious etiologies included varicella-zoster (Ramsay Hunt syndrome) (*n* = 12), neuroborreliosis (*n* = 1), rickettsiosis (*n* = 1), and elevated infection parameters in the CSF without demonstrated pathogen (*n* = 5). The remaining 424 patients were diagnosed with idiopathic facial palsy, including 14 pregnant patients. Demographics and clinical patient characteristics, per category, are summarized in Table 1. 

### 3.2. Electrophysiological Examinations

**Figure 1:**
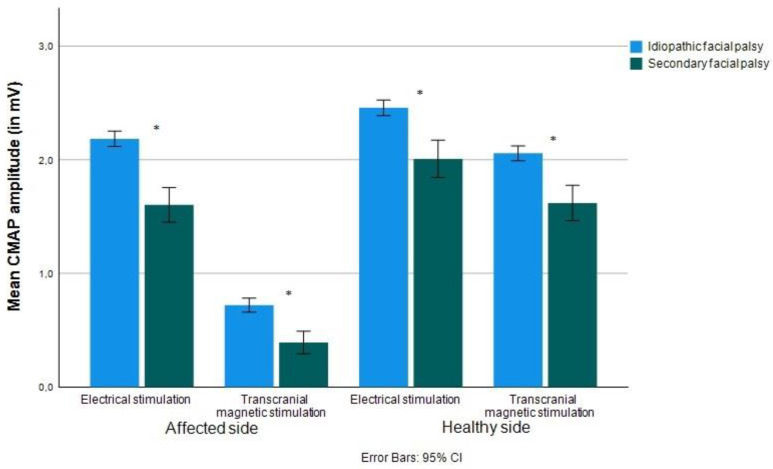
Mean CMAP amplitudes (in mV) evoked by TMS and ES of the facial nerve on the affected and healthy side. Error bars display confidence intervals. * significant at 0.05 level.

Symptom duration at the time of electrophysiological examination did not differ between the two groups (*p* = 0.895). The mean CMAP-TMS and CMAP-ES are illustrated in Figure 1. All CMAP amplitudes were significantly smaller in secondary facial palsy on both the healthy and affected side.

#### 3.2.1. Amplitude Reduction over the Facial Canal

An ARFC, as a measure of a lesion of the facial nerve within the facial canal, was observed in idiopathic and secondary palsy. The ARFC in the affected side was significantly greater in patients with secondary facial palsy ≤7 days after symptom onset (*p* <0.01). The difference in ΔARFC between both groups did not reach statistical significance (*p* = 0.076). Table 2 summarizes these findings.

#### 3.2.2. Assessment of Model Performance in Differentiating between Etiologies

To assess the ability of ARFC and ΔARFC to discriminate idiopathic from secondary facial palsies, ROC curves were calculated. The AUC for ARFC and ΔAFRC were 0.603 and 0.565, respectively, exhibiting low accuracy in discriminating idiopathic from secondary facial palsy.

**Figure 2:**
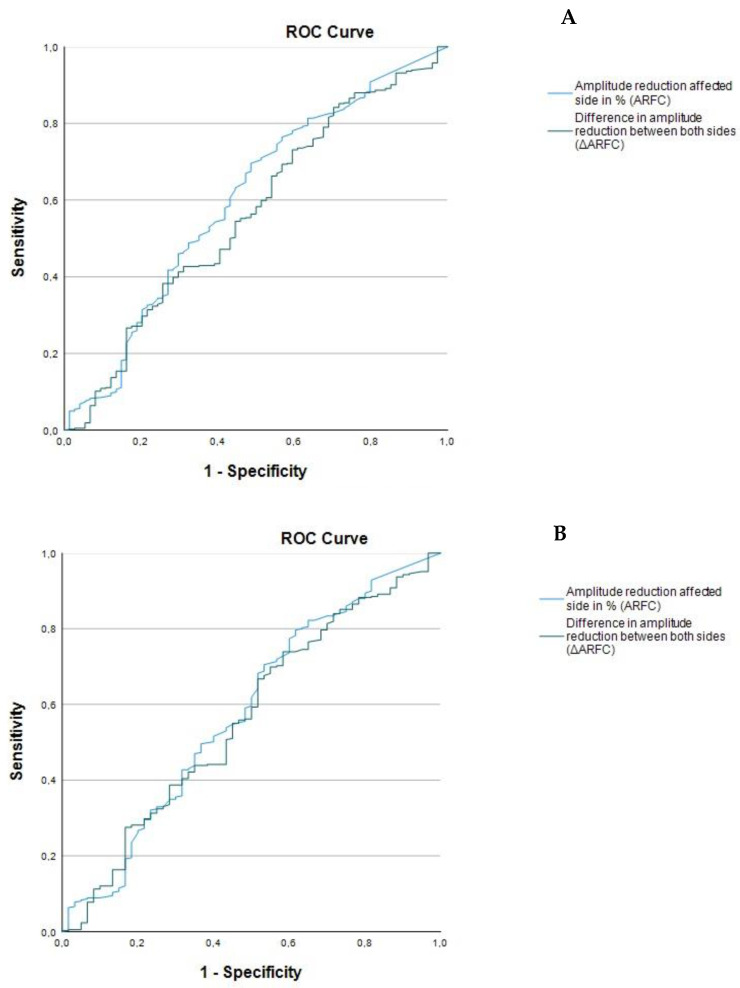
Receiver operating characteristic (ROC) curves to evaluate facial electroneurography for the diagnosis of idiopathic facial nerve palsy. (**A**) ≤7 days after symptom onset. (**B**) ≤3 days after symptom onset.

To account for the effects of disease duration on ARFC, ROC curves were constructed on a subgroup of patients with symptom duration of ≤3 days (*n* = 349 idiopathic and *n* = 60 secondary facial palsy). This did not improve the discriminative ability, as AUCs were 0.580 and 0.563 for ARFC and ΔARFC, respectively (Figure 2). 

#### 3.2.3. Electrical Stimulation and Distal Nerve Function

To study the effect of the disease duration on distal nerve function, symptom duration was correlated with CMAP-ES amplitudes. CMAP-ES on the affected side was expressed as percentage of the healthy side (relative CMAP-ES). In idiopathic facial palsy, there was a poor negative but significant correlation (r(424) = −0.207, *p* < 0.001), while there was no significant correlation in patients with secondary facial palsy (r(74) = −0.161, *p* = 0.169). 

In idiopathic facial palsy, the relative CMAP-ES was significantly lower when measured on days 4–7 after symptom onset compared to days 1–3 (*p* < 0.01), while in secondary facial palsy no significant difference between the two periods was found (Table 3). Comparing the relative CMAP-ES among patients with idiopathic and secondary facial palsy, there was no significant difference for either the first three days (*p* = 0.105) and days 4–7 (*p* = 0.324).

## 4. Discussion

### 4.1. Major Study Findings on the Value of TMS as an Early Diagnostic Tool

Our data demonstrate that an ARFC can be found in both idiopathic and secondary facial palsy and is not greater in idiopathic facial palsy. ROC curve analysis showed a very poor diagnostic value of AFRC to discriminate between idiopathic and secondary facial palsy. Previous smaller studies yielded conflicting results. Some suggested TMS is useful in discriminating between etiologies [8,9], while others found the opposite [7]. Our study, based on a large cohort of patients, is in line with the results of one larger study including *n* = 216 patients [10] and confirms that a decreased muscle response evoked by TMS cannot reliably be used to differentiate between idiopathic and secondary facial palsy and is not specific for idiopathic facial palsy. To investigate whether this method might be more suitable in a homogenous group of patients with secondary facial palsy, we assessed the diagnostic accuracy in the largest homogenous group of patients in our cohort (diabetes mellitus, *n* = 44). However, the diagnostic accuracy was still low (AUCs of 0.648 and 0.632 for ARFC and ΔARFC, respectively).

However, it was suggested that this method could only be reliably used within the first three days after symptom onset [9], as axonal degeneration might start to play a role as the disease progresses. Based on this assumption, we constructed additional ROC curves using the data of patients with a symptom duration of ≤3 days. ROC curves were similar to the original patient population, illustrating poor diagnostic accuracy of TMS in diagnosing idiopathic facial pals, regardless of the timing of the investigation.

### 4.2. Electrical Stimulation and Distal Nerve Dysfunction

Previous literature [8,9] found smaller CMAP-ES in patients with Ramsay Hunt syndrome than in patients with idiopathic facial palsy and interpreted this as being due to axonal degeneration. It was argued that extended axonal damage might be an early feature of herpes zoster infections [8,9]. We also found smaller CMAP-ES in our heterogeneous group of patients with secondary facial palsy. As the mean age in the symptomatic group was significantly higher, this might not be a result of axonal degeneration but the effect of aging on CMAP amplitude [12], as the relative CMAP-ES was not significantly lower compared to idiopathic facial palsy.

In idiopathic facial palsy, it has been suggested that axonal degeneration develops in a later stage [13,14]. Similarly, we found a small but significant decrease of relative CMAP-ES with time in idiopathic facial palsy which was not seen in secondary facial palsy. However, this decrease was small, and we consider it a pure statistical effect due to the large sample size and not clinically meaningful.

### 4.3. Limitations

This study has potential limitations. The patients in our study population are classified as idiopathic or secondary based on the current standards of practice. Thus neuroimaging and/or CSF analysis were performed only in patients with high suspicion of a secondary etiology, and some diagnoses might initially be missed. To address this, a follow-up visit after one week was established to evaluate disease progression and re-evaluate the initial diagnosis. However, some patients with secondary facial palsy might have had a non-progressive self-limiting course of the disease or responded to steroids and, therefore, might have been missed. Future studies might try to improve the diagnostic accuracy by recording the motor responses of several facial muscles, such as mm. orbicularis oculi and mentalis. Additionally, the short follow-up did not allow for the identification of electrophysiological parameters with prognostic significance, which might be an interesting topic for future research. Lastly, gender was not equally distributed between the groups. Analysis of gender-disaggregated data was not conducted as it is assumed that gender does not influence the CMAP amplitudes in electrophysiological examinations [15].

## 5. Conclusions

Our data demonstrate that the combination of ES and TMS does not reliably differentiate idiopathic from secondary facial palsy in patients with acute onset unilateral facial palsy. A reduction in CMAP amplitude evoked by TMS in relation to the amplitude evoked by ES is not specific to idiopathic facial palsy. 

## Figures and Tables

**Table 1:** Demographics and clinical patient characteristics.

Diagnosis	Secondary	Idiopathic	*p*-Value
Number of patients	74 (14.9%)	424 (85.1%)	
Gender (male/female)	48 (64.9%)/26 (35.1%)	220 (51.9%)/204 (48.1%)	<0.05 *
Age in years (±SD)	60.9 (±18.2)	45.7 (±16.6)	<0.01 *
Duration of symptoms in days (±SD)	2.6 (±1.2)	2.6 (±1.1)	0.895
Median disease severity in HB-grade (IQR)	3.00 (IQR 2–4)	3.00 (IQR 2–4)	0.373

SD = Standard Deviation. IQR = Interquartile Range. *p*-values are according to the Mann–Whitney test for the values age, duration of symptoms and severity, and the Fisher’s exact test for the parameter gender. * significant at 0.05 level.

**Table 2:** Mean values of amplitude reductions over the facial canal.

Variable	Idiopathic (*n* = 424)	Secondary (*n* = 74)	*p*-Value
ARFC on the affected side (in %) (±SD)	68.4 (±24.2)	75.3 (±25.2)	<0.01 *
ARFC on the healthy side (in %) (±SD)	16.8 (±11.9)	20.1 (±15.8)	0.245
ΔARFC (in %) (±SD)	51.6 (±25.6)	55.2 (±30.8)	0.076

* significant at 0.05 level. Amplitude reduction over the facial canal (ARFC) was calculated as 100—(CMAP evoked by TMS/CMAP evoked by electric stimulation * 100%). Delta amplitude reduction over the facial canal (ΔARFC) was calculated as the ARFC on the affected side—the ARFC on the healthy side, and is the absolute difference between the two percentages.

**Table 3:** Mean values of CMAP amplitudes evoked by electrical stimulation.

Variable	Idiopathic, ≤3 Days (*n* = 349)	Idiopathic, >3 Days (*n* = 75)	*p*-Value	Secondary, ≤3 Days (*n* = 60)	Secondary, >3 Days (*n* = 14)	*p*-Value
CMAP-ES affected side in mV (±SD)	2.24 (±0.69)	1.91 (±0.73)	<0.01 *	1.64 (±0.65)	1.43 (±0.67)	0.350
CMAP-ES healthy side in mV (±SD)	2.46 (±0.72)	2.43 (±0.71)	0.797	2.02 (±0.73)	1.94 (±0.64)	0.868
Relative CMAP-ES in % (±SD)	92.78 (±21.58)	79.04 (±23.29)	<0.01 *	86.97 (±42.23)	72.33 (±23.11)	0.224

Comparison of the CMAP amplitudes evoked by electrical stimulation (CMAP-ES) ≤3 days and >3 days after symptom onset, separated per etiology, and for the CMAP-ES on the affected side as percentage of the CMAP-ES on the healthy side (relative CMAP-ES). SD = Standard deviation. * significant at 0.01 level.

## Data Availability

The data presented in this study are available on request from the corresponding author. The data are not publicly available due to ethical restrictions.
